# Early differentiation of neurodegenerative diseases using the novel QSM technique: what is the biomarker of each disorder?

**DOI:** 10.1186/s12868-022-00725-9

**Published:** 2022-07-28

**Authors:** Farzaneh Nikparast, Zohreh Ganji, Hoda Zare

**Affiliations:** 1 Medical Physics Research Center, Mashhad University of Medical Sciences, Mashhad, Iran; 2 Department of Medical Physics, Faculty of Medicine, Mashhad University of Medical Sciences, Mashhad, Iran

**Keywords:** Quantitative susceptibility mapping, Beta-amyloid PET, Alzheimer’s disease, Atypical primary Parkinsonism, Parkinson’s disease

## Abstract

During neurodegenerative diseases, the brain undergoes morphological and pathological changes; Iron deposits are one of the causes of pathological changes in the brain. The Quantitative susceptibility mapping (QSM) technique, a type of magnetic resonance (MR) image reconstruction, is one of the newest diagnostic methods for iron deposits to detect changes in magnetic susceptibility. Numerous research projects have been conducted in this field. The purpose of writing this review article is to identify the first deep brain nuclei that undergo magnetic susceptibility changes during neurodegenerative diseases such as Alzheimer's or Parkinson's disease. The purpose of this article is to identify the brain nuclei that are prone to iron deposition in any specific disorder. In addition to the mentioned purpose, this paper proposes the optimal scan parameters and appropriate algorithms of each QSM reconstruction step by reviewing the results of different articles. As a result, The QSM technique can identify nuclei exposed to iron deposition in various neurodegenerative diseases. Also, the selection of scan parameters is different based on the sequence and purpose; an example of the parameters is placed in the tables. The BET toolbox in FSL, Laplacian-based phase-unwrapping process, the V_SHARP algorithm, and morphology-enabled dipole inversion (MEDI) method are the most widely used algorithms in various stages of QSM reconstruction.

## Keypoints

1. In this article, A review of the results of articles on the use of QSM technique to identify nuclei exposed to iron deposition in various neurodegenerative diseases was performed.
2. Brain nuclei with the highest changes in iron deposition were identified as a biomarker for the identification of specific neurological diseases
3. By studying recent articles, The best toolbox for each step of the QSM processing algorithm was introduced.

## Introduction

The presence of iron is the basis of many biological functions in the body, such as cell growth, cell differentiation, proper enzymes function, etc. [1]. However, according to the evidence, increased iron deposition leads to brain cells damage and dysfunction of neurons [2]. In various neurodegenerative diseases, such as Alzheimer's Disease (AD) and Parkinson's Disease (PD), iron deposition in the deep gray nuclei of the brain has been proven [3]. The magnetic susceptibility of the brain reflects the components of the tissue; different areas of the brain are exposed to these susceptibility changes due to iron deposition [4]. As one of the most advanced and modern imaging methods, magnetic resonance imaging (MRI) has various techniques to examine these changes, such as T2 * weighted imaging (T2*WI), susceptibility-weighted imaging (SWI), relaxation rates (R2*), and field-dependent relaxation rate increase (FDRI) [5]. Despite their relatively good performance, there are some drawbacks to using each. Almost all the methods mentioned suffer from the Blooming Artifact problem. Besides that T2 * WI depends on the direction and parameters of scanning; measurements obtained in the SWI method are non-local; R2 * method depends on water and iron contents; and for performing the FDRI method, two magnetic fields with two different strengths are required [6, 7].

Today, a new, non-invasive method based on the magnetic susceptibility properties of tissues has been introduced called Quantitative susceptibility mapping (QSM), which does not have many of the limitations of previous methods [8, 9]. Different sequences are used to perform this reconstruction.

The QSM technique is based on the identification of depositions that change the magnetic susceptibility of the tissue by the processing of magnitude and phase images obtained from multi-echo sequences.

Researchers use different algorithms to perform each of the processing steps.

**Fig. 1:**
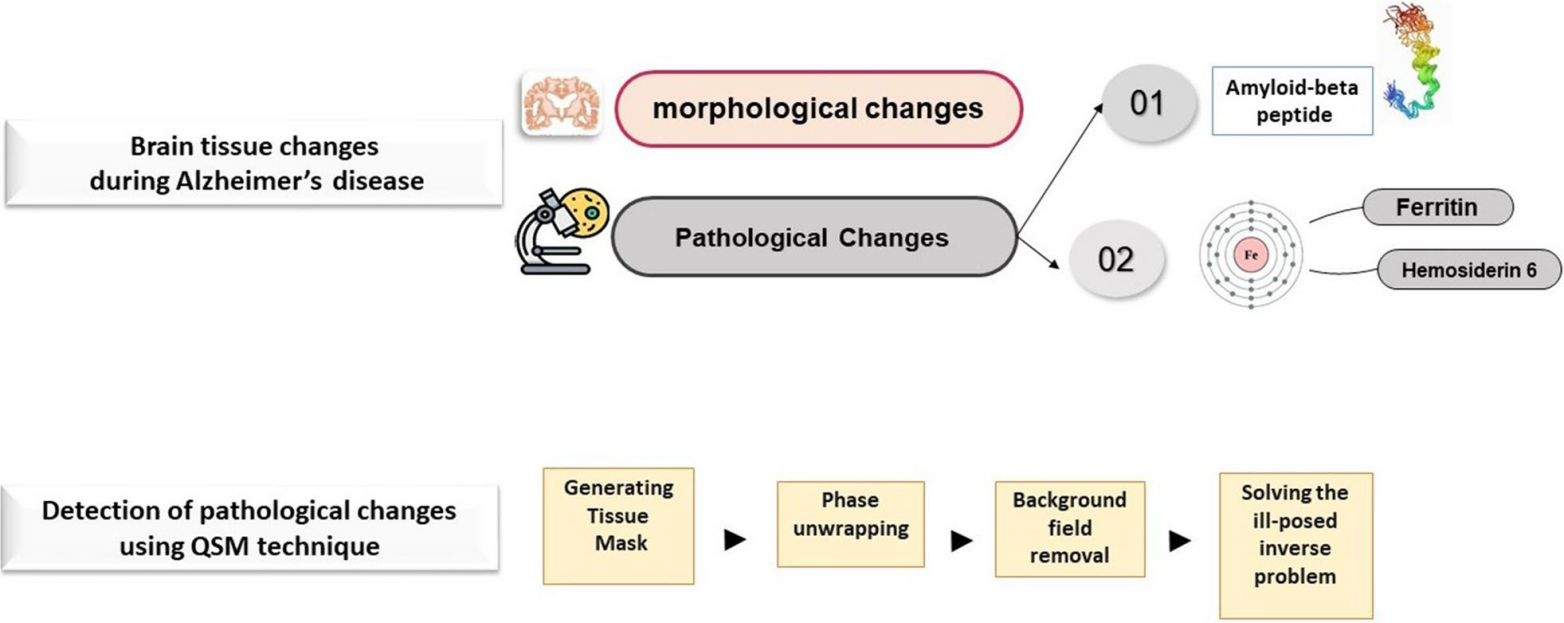
Diagram the types of brain changes during Alzheimer's disease and the stages of QSM reconstruction.

First, the Generating Tissue Mask from the magnitude images is performed. Then the three steps of Phase unwrapping, Background field removal, and solving the ill-posed inverse problem, respectively, run on phase images (Fig. 1).

**Fig. 2:**
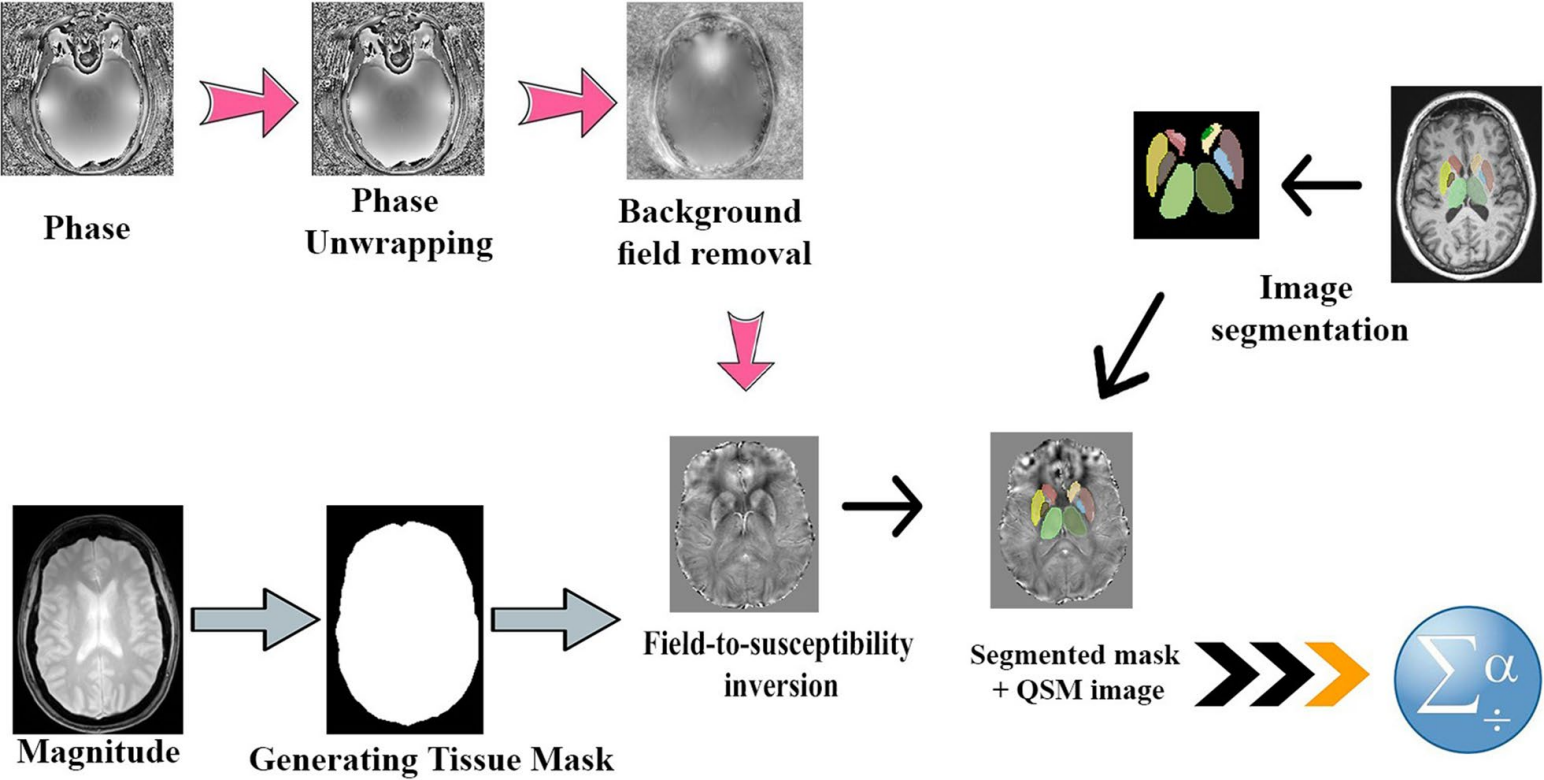
Steps of QSM reconstruction, segmentation and segment analysis

To identify different areas of the brain, tissue segmentation is performed manually or automatically.

Finally, QSM values can be evaluated with the help of various software such as 3D Slicer.

QSM is preferable to the R2 ∗ or FDRI methods for evaluating the amount of iron deposition in the brain.

QSM has advantages over methods such as R2^∗^ or FDRI, such as: fixing the blooming artifacts, and not depending on echo-time, water content, or field strength.

However, multi-echo Gradient echo (mGRE) is the most common sequence used in QSM reconstruction [10].

One of the essential concerns of researchers is to select the appropriate sequence and parameters of MRI scans for QSM reconstruction.

In this research, while introducing the different stages of this reconstruction, the appropriate algorithms will be introduced based on the summary of previous research projects.

Also, some problems with the QSM technique and tricks to deal with it will be discussed; this research approach has not been made in previous studies.

In the following, changes in the magnetic susceptibility of different brain nuclei in each disease will be introduced based on the findings of other studies, which can be used in the clinical field.

## Material and methods

PRISMA recommendations were used in selecting relevant studies to write this systematic article (Table 1).

Databases PubMed and Google Scholar were used to search for articles on March 21, 2021.

The following search terms include (Quantitative susceptibility mapping) AND MRI AND (Alzheimer diagnosis with QSM) AND (Parkinson diagnosis with QSM) AND (Alzheimer diagnosis with PET). Moreover, the time frame was set between 2013 and 2021 to focus on the latest findings (360 results). Then, the titles and abstracts of all these articles were screened; unrelated ones excluded and the full text of the remaining articles reviewed. Criteria for selecting articles include 1. Research about changes in the magnetic susceptibility of deep gray nuclei during Alzheimer's or Parkinson's disease and 2- Mention of imaging sequence parameters performed; and finally, 30 articles selected.

## Results

The QSM technique is a new method for mapping altered areas of magnetic susceptibility in the brain that the output map is magnetic susceptibility in part per million (ppm).

It is a kind of post-processing technique applied to the magnitude and phase images of echo gradient sequences usually. However, most processing operations are performed on phase images [11–13].

## QSM reconstruction steps

QSM reconstruction has several steps: Generating Tissue Mask, Phase unwrapping, Background field removal, and solving the ill-posed inverse problem (Fig. 2).

### Generating tissue mask

It is essential to choose the correct brain mask in border areas, especially near the air-tissue or bony junctions, because signal loss happens at the brain boundaries in the magnitude image due to susceptibility differences in these regions. So it is necessary to remove the noisy regions in the GRE phase images. This step is essential to define the region of interest (ROI) for background field removal and QSM step. Usually, we can use a brain extraction tool (BET) in FMRIB Software Library to generate a brain mask [14].

### Phase unwrapping

The GRE signal phase of the MRI detects only the phase values in the range [− π, π], but QSM algorithms require the phase range [− 2π, 2π] for the reconstruction process.

Aliasing artifact occurs when the sampling is less than the allowable limit; the result of this artifact is the appearance of black-and-white bands called wraps. For correct estimating the magnetic field turbulence, phase unwrapping is required, which is aliasing removal of the phase data.

The spatial domain can be done using the conventional path-based or Laplacian-based unwrapping algorithms [15] and linear fitting methods in the temporal domain [16].

### Background field removal

After phase unwrapping, magnetic field inhomogeneity induces the background component because of the air–tissue and air-bone interfaces. To extract the local field induced by the local susceptibility distribution, these unwanted fields must be removed.

Techniques for background removal include:

1. The harmonic phase removal using a Laplacian operator (HARPARELLA) and sophisticated harmonic artifact reduction for phase data (SHARP) and its species like regularization enabled SHARP (RESHARP) [17, 18].
2. Projection onto dipole fields (PDF) [19]**.**
3. By assuming a boundary value known as the Laplacian boundary value (LBV) method and solving the Laplacian equation [20].

Removal of low-frequency spatial components in the texture is necessary for accurate QSM inversion; hence high-Pass filtering can be used.

### Field-to-susceptibility inversion

One of the problems during QSM reconstruction is the inverse problem that can be solved in several ways:

1. Susceptibility calculation through multidirectional sampling (COSMOS) is one of the first effective methods that has been introduced [21].It was the gold standard in QSM because the resulting susceptibility map had no streaking artifacts [22].However, this method requires different anatomical orientations and a long scan time, so it is not a good suggestion for in vivo clinical studies. Also, we can use several solutions that are more practical than COSMOS to reduce streaking artifacts like:
2. Iterative image space-based optimization tactics:
  This algorithm uses iterative methods such as steepest descent (SD) and conjugate gradient to solve the inverse problem. Nevertheless, there are differences between the assumed mathematical properties and the physical reality, so errors occur in susceptibility reconstruction.
3. To solve the problem mentioned, we can use morphology-enabled dipole inversion (MEDI) method. This toolbox is a collection of MATLAB (MathWorks, Natick, MA) routines for reconstructing the QSM and uses the appropriate anatomical information contained in the magnitude images [23].
4. Structural feature-based collaborative reconstruction (SFCR) is an algorithm recommended by Bao, L., et al. in 2016 to recover the structure edges and tiny features and decrease noise and artifact issues because the anatomy seen in the magnitude and phase images does not always correspond to the resulting QSM map [24].
5. Homogeneity-enabled incremental dipole inversion (HEIDI) is another appropriate method for QSM reconstruction that uses the combined edge information derived from both the magnitude and phase images [25].
6. Another method is deep neural network reconstruction techniques known as Deep QSM [26, 27]or QSMnet [28], introduced to solve the inversion problem. These deep neural networks generate high-quality susceptibility maps from single orientation data and perform impressively compared to iterative methods [29]. The fully convolutional deep neural network has been used in Deep QSM to develop artifact-free susceptibility maps. This method offers superior image quality than thresholded K-space-Division (TKD) or MEDI using the modified U-net structure.

### Image segmentation

After QSM reconstruction, segmentation is required to quantify the region of interest (ROIs) values. Segmentation is done on high-resolution images such as T1-weighted images usually.

ROIs segmentation is possible in two ways:

1. Manually target areas definition, which takes a long time and depends on the operator.
2. Automatic brain mapping to existing brain atlases: commonly based on T1 [30].

## QSM problems and tricks to solve them

### Relatively long time

QSM processing is often applied to multi-echo Gradient echo sequence (mGRE). One of the problems with this sequence is the relatively long data acquisition time. This time is not suitable for patients who cannot hold their heads for a few minutes. Based on the results of various articles, tricks can be used to solve this problem for a relatively long time:

#### Single-shot EPI-QSM method

Based on the results of research by Wei, H., et al. in 2017 [31], 2D echo-planar imaging (EPI) can be used for rapid reconstruction of QSM, like using functional QSM (fQSM) at 7 T and 9.4 T, because it has a high temporal resolution. In other words, using single-shot EPI-QSM, subcortical gray matter susceptibility can be measured at minimal scan time. The use of this method in a standard clinical system is optimal [32].

Sun and Wilman in 2015 performed ROIs analysis; the results showed a high linear correlation between the iron concentration in the subcortical gray matter (GM) and EPI-QSM; they also demonstrated that the susceptibility was statistically equal to the standard QSM echo-gradient [32]. However, there are phase errors in QSM reconstruction from 2D EPI data. Integrating two-dimensional phase correction and removing the three-dimensional background phase is an excellent way to solve this problem. In other words, with the joint 2D and 3D phase processing of 2D EPI data and improved susceptibility reconstruction algorithm, susceptibility images with the desired quality can be obtained. It is a simple 2D + 3D phase-processing technique for QSM based on 2D GRE-EPI data, and the results of 8-s scan time on the 3T system by this method are similar to 3D mGRE QSM [31].

#### Using 3T MRI scanner system

In 2020, Spincemaille, P., et al. concluded that it is possible to obtain QSM images with the same quality obtained in the 3 T MRI scanner with half the time by the 7T MRI scanner [33].

### Relatively low-quality QSM images

#### Using multi-atlas quantification tool

In neuroscience and neuroimaging techniques, Magnetic susceptibility human brain multi-atlas quantification is a valuable tool for automatic segmentation and quantification of QSM-based magnetic susceptibility measures.

It has acceptable accuracy and reliability; these atlases facilitates QSM analysis [34].

Although QSM is a powerful method, it is better not to use a slice thickness greater than 2 mm to prevent the susceptibility reconstruction of smaller structures like the dentate nucleus (DN), red nucleus (RN), and substantia nigra (SN) bias based on slice thickness [35].

## Regions subject to magnetic susceptibility changes during neurodegenerative disease

Based on the findings of Li et al. in 2021, during the aging process, iron volume decreases in all structures except SN and DN. The pattern of iron deposition in the deep gray nuclei of the brain is different in various diseases and situations [35].

### Basal ganglia

Basal ganglia (BG) areas, where iron deposition occurs slowly, are areas where any changes usually lead to a wide range of neurological and mental illnesses [36, 37]. BG contains the highest amount of iron in the brain, which is composed of the substantia nigra (SN), putamen (Pu), globus palidus (GP), subthalamic nucleus (STN), and caudate nucleus (CN) [37, 38].

### Internal capsule

The BG connecting fiber tract internal capsule (IC) is a white matter (WM) myelin structure that attaches to and passes through the BG and is divided into several structures, including the posterior limb of the internal capsule (PLIC) and the anterior limb of the internal capsule (ALIC) [39].

It is anatomically located between the thalamus and the CN medially and between the lentiform nucleus (PU and GP) laterally.

### Hippocampus and Fimbriae

The hippocampus is a particular cortical tissue (gray matter) in the temporal lobe. It is one of the first areas affected in the early stages of Alzheimer's [40].

Fimbriae are a small bundle of WM fibers located along the upper surface of the hippocampus. They are part of the central WM system attached to other limbic system structures [41]. It can be said that fimbriae are a structural bridge between different structures of the brain and hippocampus and are critical in the function of memory and the hippocampus [42].

## Use of QSM technique in the diagnosis of Alzheimer's disease

As AD progresses, different brain areas are affected by changes in magnetic susceptibility; QSM is an excellent way to diagnose AD in the early stages due to evaluation the pattern of iron accumulation in the brains.

A study was conducted in 2019 by Gong et al. to prove the fundamental principle that the QSM technique can detect diamagnetic materials such as beta-amyloid plaques [43].

The cylindrical phantom used in this exam had five straws which contained gadolinium, beta-amyloid buffer only, and beta-amyloid with buffer solution.

In the end, beta-amyloid transgenic mouse models were scanned to evaluate their deep gray nuclei magnetic susceptibility changes. The hypothesis was that amyloid-beta accumulation increases electron density and causes significant changes in local susceptibility.

These changes are significant enough to cause contrast to the surrounding tissues and can be seen using MRI quantitative susceptibility mapping (QSM). Finally, they showed that the diamagnetic susceptibility of amyloid-beta could be demonstrated by this method. Detection and evaluation of noninvasive beta-amyloid accumulations by QSM- MRI is a significant step in the early and rapid diagnosis of Alzheimer's Disease and the prevention of disease progression with appropriate and timely drugs or other therapies [44].

Now, we express the results that indicate the accuracy and sensitivity of the QSM technique; this technique shows more differences between various groups of cognitive disorders than other post-processing methods.

To prove this, Kim et al. researched in 2017 on the efficiency of gray matter volume (GMV) and QSM method in detecting differences between control, amnestic mild cognitive impairment (aMCI), and AD individual groups [45].

Susceptibility differences in known areas of iron and β-amyloid accumulation were more remarkable in individuals in the normal cognition, aMCI, and AD groups than GMV changes.

A study was conducted in 2020 by Spotorno, N., et al. to prove the accuracy of the results of the QSM technique [46]; advanced imaging techniques such as QSM and tau-positron emission therapy (tau-PET) were used to investigate the relationship between iron accumulation and abnormal tau accumulation in AD.

According to the results, in some regions affected by AD, there is an increase in iron content and tau-PET signal.

Quantitative susceptibility precisely conforms to tau-PET results. There is an excellent relationship between quantitative susceptibility values and tau-PET in younger participants.

As a result, this method is sensitive to iron load and, according to the accuracy of its results, can be used to study the disease process.

These in vivo results provide evidence of an association between iron deposition, tau accumulation, and nerve damage that enhances our understanding of the role of iron in the progression of AD.

### Areas prone to changes in magnetic susceptibility during Alzheimer's disease

**Fig. 3:**
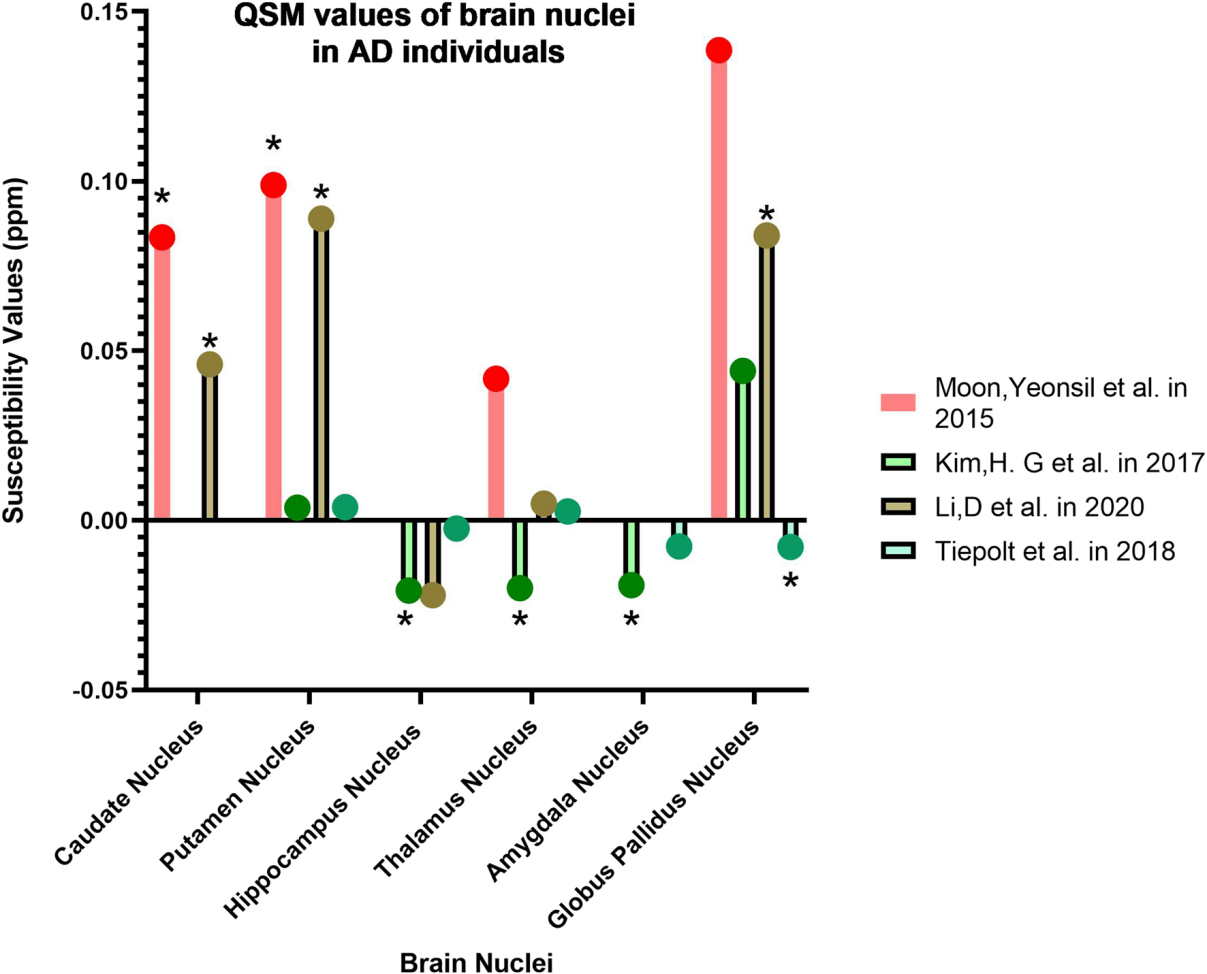
Changes in the magnetic susceptibility of brain nuclei in Alzheimer's disease (asterisk: has a significant difference in QSM values compared to the control group)

**Fig. 4:**
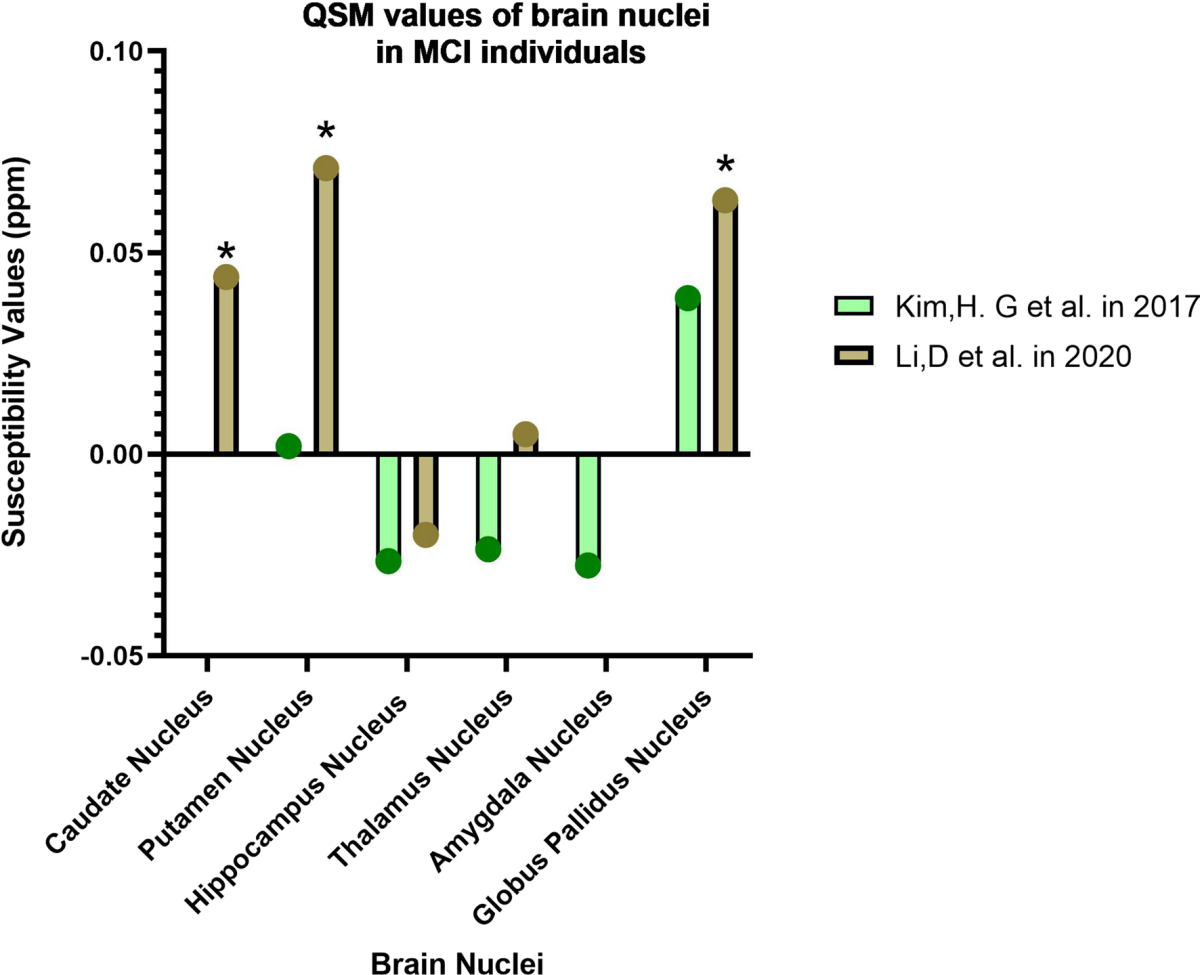
Changes in the magnetic susceptibility of brain nuclei in MCI (asterisk: has a significant difference in QSM values compared to the control group)

**Fig. 5:**
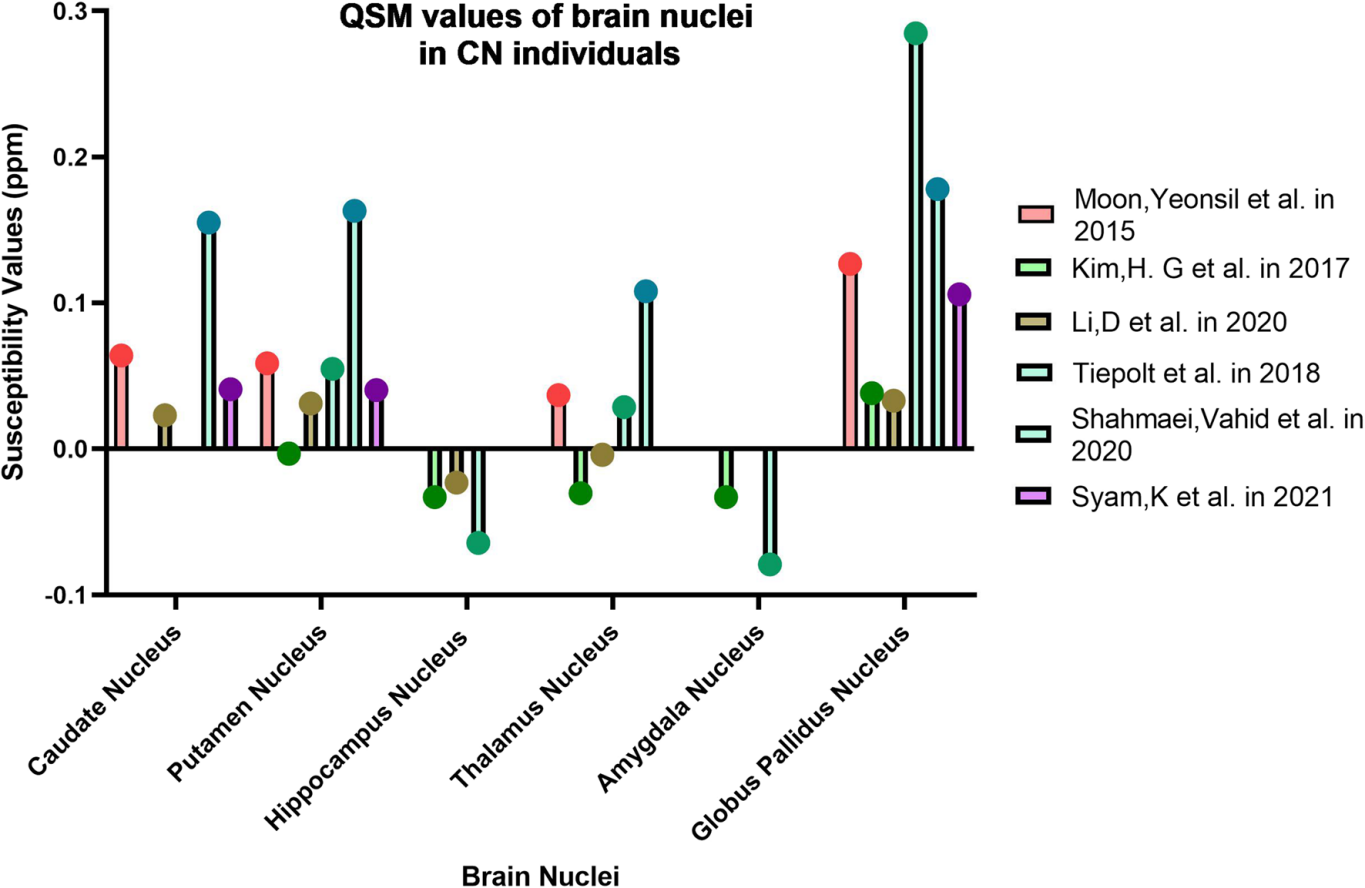
Changes in the magnetic susceptibility of brain nuclei in CN (asterisk: has a significant difference in QSM values compared to the control group)

In this section, we introduce the areas introduced as biomarkers of different stages of AD based on the results of some articles (Tables 2, 3, 4, 5; Figs. 3, 4, 5).

**Table 1:**
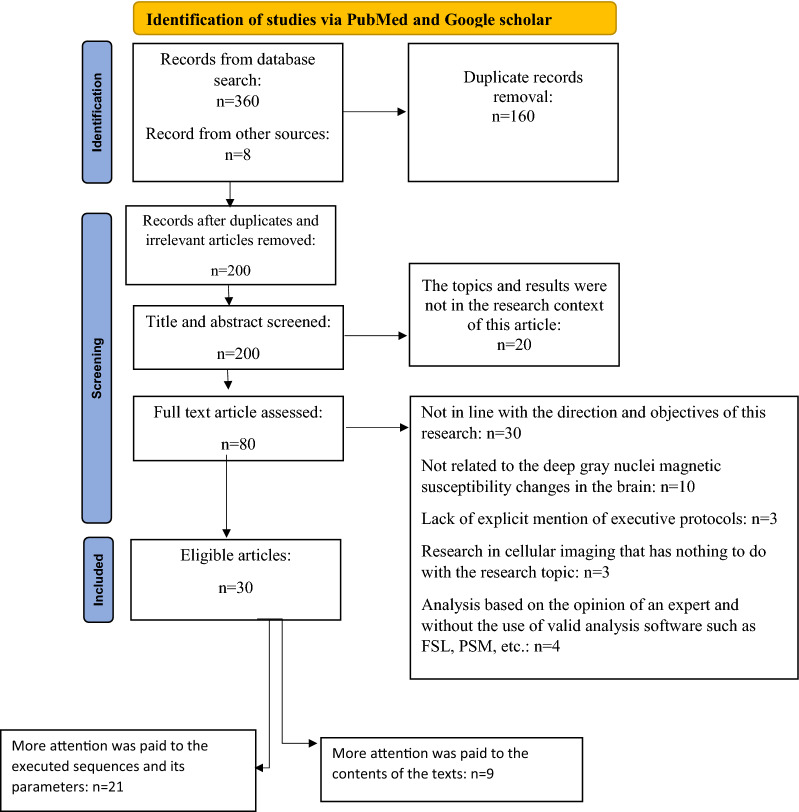
PRISMA recommendations for selecting studies related to the objectives of this article

**Table 2:**
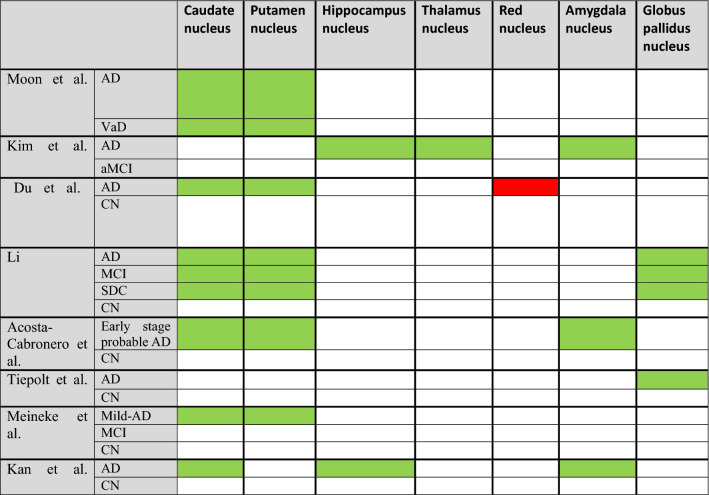
Changes in the QSM values of the brain nuclei in AD and MCI

Green = increase QSM values, Red = Decrease QSM values

**Table 3:** The QSM values and MMSE scores correlation in AD

		Caudate nucleus	Pallidum
Du et al. [5]	AD	R = − 0.52, P < 0.01	
	CN		
Tiepolt et al. [75]	AD		R = − 0.69, P = 0.001
	CN

**Table 4:** The QSM values and age correlation in AD and MCI

		Caudate nucleus		Putamen nucleus		Dentate nucleus		Globus pallidus nucleus	Hippocampus nucleus	Thalamus nucleus
		R	L	R	L	R	L			
Moon et al. [47]	AD	*R* = − 0.291, *P* = 0.031								
	CN	*R* = 0.532, *P* = 0.023		*R* = 0.678, *P* = 0.002						
Du et al. [5]	CN	R = 0.43, P = 0.019		R = 0.68, P = 0.0000	R = 0.67, P = 0.0001	R = 0.54, P = 0.0026	R = 0.72, P = 0.0000			
Li et al. [3]	AD			R = *0.658, *P = *0.001*				R = *0.636, *P = *0.001*		
	MCI							*R* = 0.531, *P* = 0.011	*R* = 0.516, *P* = 0.014	
	SCD									R = 0.421, *P* = 0.036

**Table 5:** The results of receiver operating characteristic curve (ROC) test in AD and MCI

		Caudate nucleus	Globus pallidus nucleus	Putamen nucleus	Hippocampus nucleus	Thalamus nucleus	Amygdala nucleus
Kim et al. [45]	AD				AUC = 0.803, P < 0.0001	AUC = 0.742, P = 0.0036	AUC = 0.831, P < 0.0001
	aMCI				AUC = 0.709, P = 0.0189	AUC = 0.692, P = 0.0286	
	AD and aMCI						AUC = 0.739, P = 0.0044
Li et al. [52]	AD	AUC = 0.84, P < 0.0001	AUC = 0.99, P < 0.0001	AUC = 0.96, P < 0.0001		AUC = 0.69, P = 0.0137	
	MCI	AUC = 0.81, P < 0.0001	AUC = 0.89, P < 0.0001	AUC = 0.94, P < 0.0001		AUC = 0.67,P = 0.02	
	SDC	AUC = 0.71, P = 0.0069	AUC = 0.81,P < 0.0001	AUC = 0.90, P < 0.0001			
Meineke et al. [72]	AD	AUC = 0.86, P = 0.016		AUC = 0.94, P = 0.013

Evaluation of the difference in magnetic susceptibility of deep brain nuclei between AD and vascular dementia (VaD) patients was performed in 2016 by Moon et al.; researchers concluded that patients with VaD and AD have more iron deposition in the Putamen and caudate nucleus [47].

However, more study conducted in 2018 by Du et al. [5]; according to the results, unlike bilateral RN, which has lower susceptibility values in AD than the controls, the susceptibility of bilateral CN and Pu in patients with AD was significantly higher than the control group.

There is a significant relationship between decreased Mini-mental state examination (MMSE) scores and Montreal cognitive assessment (MoCA) scores and increased magnetic susceptibility on the left CN. (MMSE and MoCA: two widely used questionnaires for the evaluation of cognitive impairment in AD patients).

Their study showed that the amount of left CN magnetic susceptibility could be introduced as an indicator of disease severity in mild to moderate AD.

During AD progression, iron deposition in the BG and decreased blood perfusion were observed in the target areas [48].

QSM values in deep and inferior gray nuclei, especially the Putamen and pallidus, can be introduced as a cognitive biomarker. QSM values in Putamen can be used as an imaging biomarker for early detection of AD [8, 48].

In addition to examining the susceptibility of deep GM nuclei, Fimbria can be assessed.

Au et al. researched in 2021 [49]; according to their results, Fimbria has higher magnetic susceptibility in patients with AD than the control group. AD can be diagnosed in the early stages of Disease by QSM.

WM structures attached to deep gray nuclei also undergo changes in magnetic susceptibility during AD.

Another study was performed in 2020 by Pu, R., et al.to investigate the effect of iron deposition on the myelin development of the surrounding area [50].

They evaluated brain iron concentrations in BG regions, including CN, GP, and PU of old adult and young macaques using QSM. The myelin water fraction (MWF) technique was also used to measure the myelin content of BG-connecting fiber tracts, including the ALIC and PLIC.

These results showed moderate to high positive correlations between BG's magnetic susceptibility and the MWF of IC structures anatomically connected to BG. So the impact of iron concentration in BG on myelin development in these anatomically connected WM structures proved.

## Use of QSM technique in the diagnosis of Parkinson's disease and a range of similar diseases

**Fig. 6:**
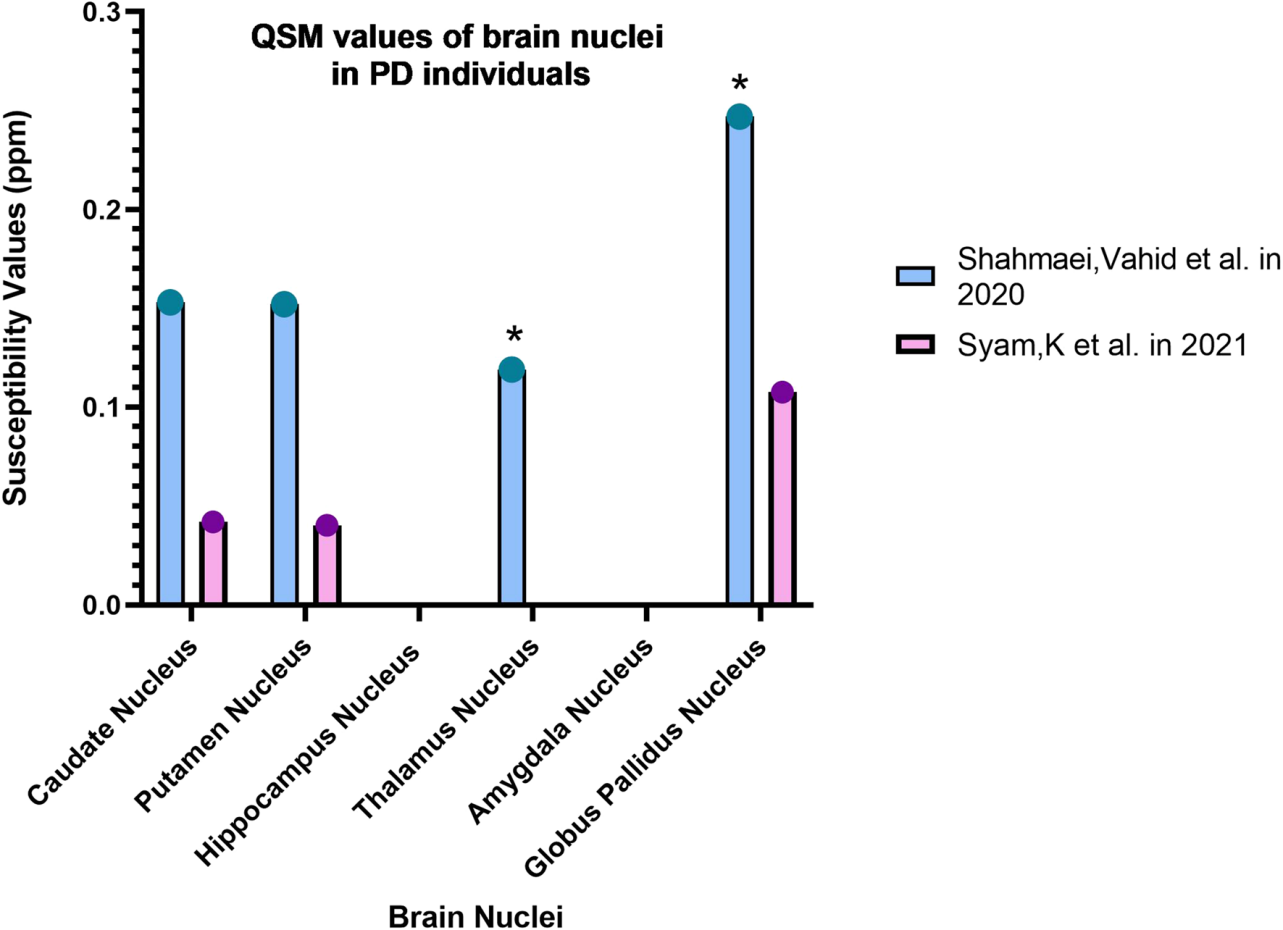
Changes in the magnetic susceptibility of brain nuclei in PD (asterisk: has a significant difference in QSM values compared to the control group)

Parkinson's Disease is another cognitive disorder that is very common after AD. Besides that, there is a range of cognitive disorders with very similar characteristics to PD. Parkinson's disease is sometimes associated with dementia and is called Parkinson's disease dementia (PDD), and sometimes the characteristics of the patient's disorder fall into the category of Atypical Parkinsonisms (APPs). APPs also include Progressive Supranuclear Palsy (PSP), which is a fatal syndrome. Biomarker identification is essential for the early detection of these disorders and differentiation from each other (Figs. 5, 6).

### Areas prone to changes in magnetic susceptibility during Parkinson's disease

Shahmaei et al. in 2019 concluded that high QSM values in Red Nucleus, Subtania Nigra, and Globus Pallidus nuclei are helpful for diagnosis and staging patients with Parkinson's disease [51] (Tables 6, 7).

**Table 6:**
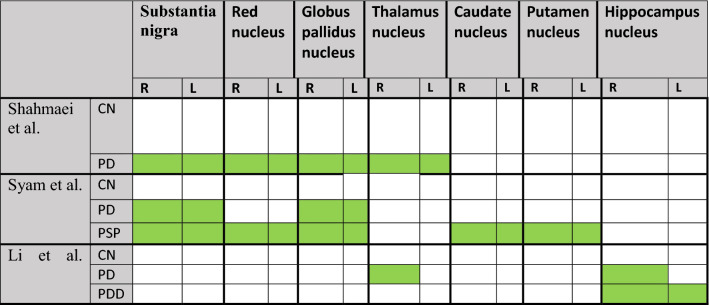
Changes in the QSM values of the brain nuclei in PD, PDD, and PSP

**Table 7:** The QSM values and disease stage correlation in PD, PSP, and PDD

		Substantia nigra	Red nucleus	Globus pallidus nucleus	Hippocampus nucleus	
					R	L
Shahmaei et al. [51]	PD	R = 0.751, P < 0.001	R = 0.538, P < 0.001	R = 0.751, P < 0.001		
Syam et al. [53]	PD	R = 0.50, P = 0.01		R = 0.39, P = 0.06		
	PSP	R = 0.37, P = 0.05	R = 0.64, P < 0.001			
Li et al. [52]	PD				R = − 0.38, P = 0.001	R = − 0.32, P = 0.006

Li et al. performed a study in 2018to find the difference in iron accumulation pattern between PD and PDD groups by QSM measurement method [52]. According to this study, higher iron deposition was observed in bilateral hippocampus patients with PDD than healthy individuals. Also, compared to non-demented patients with PD, higher iron deposition was observed in the unilateral hippocampus of patients with PDD. There is a moderate correlation between iron content and cognitive disorders in PD and patients with PDD.

In 2021, Syam conducted a research project on the difference in magnetic susceptibility values of brain nuclei between PD and PSP patients using QSM [53].

Results showed that substantia nigra mineralization was much higher in patients with progressive supranuclear palsy (PSP) than PD patients. Also, deep gray nuclei (caudate nuclei, putamen, globus pallidus, and red nuclei) had higher magnetic susceptibility values in PSP patients than healthy volunteers and PD.

The mental assessment determined a strong relationship between the intensity of deep GM mineralization and clinical severity in patients with PSP.

The most substantial relationship was related to the red nucleus in PSP patients.

Nevertheless, the correlation between substantia nigra QSM values and PD stage was moderate on the Parkinson's disease. As a result, quantitative susceptibility mapping techniques can differentiate PD from PSP and control progress [53].

A study was conducted in 2020 by Fedeli et al.; this study aimed to quantify the iron deposition and accumulation in patients with PD and APPs in the putamen, globus pallidus, red nuclei, caudate nucleus, and thalamus using QSM [54]. As a result, QSM values may help early diagnosis and differentiation between APPs. Also, during aging, the amount of QSM in globus pallidus lateralis (GPL) gradually increases, leading to better clarity and detection of globus pallidus medialis (GPM) in the elderly PD [55].

## Using QSM technique to diagnose other neurodegenerative diseases

### Huntington's disease

One of the most acute symptoms of Huntington's disease is an increase in iron depositions in the striatum, which causes free radicals and damage to neurons. There is an inverse relationship between the QSM values and the striatum's size.

On the other hand, this increase in QSM values has been observed in the putamen and caudate nuclei, which is proportional to the severity of the disease [56, 57].

According to previous research, iron deposition in the striatum nucleus has started early in the onset of Huntington's disease and can help in the early diagnosis of this disorder [58].

### Wilson disease

One of Wilson's disease's essential features is an impairing in the biliary excretion of copper, which occurs due to a mutation in hepatic copper transport protein; this disorder causes the accumulation of copper in organs such as the liver, and brain, etc. [59].

Chelation therapy can be a good treatment if it is started in time, which is necessary to identify Wilson's disease early [60].

One of the QSM image hallmarks in patients with Wilson's disease is an increase in magnetic susceptibility in the basal ganglia due to abnormal copper deposition in this area, which can act as a primary marker.

Doganay et al. Have shown that even when no signal changes are detected in T1-weighted and T2-weighted MRI images, the QSM technique shows increased susceptibility in the basal ganglia and brainstem of patients with Wilson disease, which helps in early diagnosis and start the treatment process on time [60].

### Amyotrophic lateral sclerosis (ALS)

Amyotrophic lateral sclerosis, or ALS, is a neurodegenerative disease that has a devastating effect on the brain and spinal cord nerve cells; the patient loses muscle control during this disease.

It is difficult to diagnose and is usually diagnosed after one year from the start, so early diagnosis helps patients start drug treatment and slow the progression of the disease [61].

According to pathological studies, abnormally high levels of iron in the motor cortex cause oxidative stress and the death of nerve cells [62].

The QSM technique has a much higher diagnostic accuracy than T2-weighted, T2 * -weighted, and FLAIR images to detect abnormal iron deposition in the motor cortex of these patients [63].

### Friedreich ataxia (FA)

Ataxia is a group of rare neurological diseases (diseases related to the nervous system) that affect movement. People with ataxia often have difficulty with balance, swallowing, and speech.

It is usually caused by damage to the part of the brain that conforms to the movement (the cerebellum).

Ataxia can occur at any age; it is usually progressive, meaning that it can worsen over time.

One group of ataxia disorders is Friedrich's ataxia (FA), the most common type of genetic ataxia. It usually occurs between the ages of 5 and 15. In addition to worsening movement problems, people with Friedrich's ataxia develop muscle stiffness and gradually lose the strength and sensation of their arms and legs.

Histological studies after Friedrich's ataxia have shown a reduction in the size of the cerebellar; one of the benefits of the QSM technique is that it helps to estimate the volume of these structures, accurately estimate the iron content of the brain structures, and detect the disease early.

The importance of this technique becomes clear when we consider the inability of conventional MRI images to diagnose mild and subtle cerebellar atrophy [64, 65].

### Major depression

The most important structures involved in developing major depression are the habhabenular nuclei in the diencephalon, which are engaged in learning from negative experiences and reward processing but are not easily seen in standard MRI sequences.

The advantage of the QSM technique is the display of these structures due to the formation of iron deposits in them [66].

## Association between iron deposition, amyloid-beta plaques, and neurons death in Alzheimer's disease

The mechanism of damage to neurons by iron can be investigated in two ways.

First, iron produces reactive oxygen species under normal conditions without illness and causes oxidative damage and cell death through ferroptosis [67, 68].

But the second mechanism is more specific to the disease.

In this situation, iron interacts with the hallmarks of neurodegenerative diseases such as amyloid-β (Aβ) plaques, α-synuclein aggregates, and tau protein and causes their production and accumulation.

The integration of iron in their structure further increases their oxidative properties and cell death [67, 69].

In 2015, a study was conducted by van Bergen et al., and individuals with MCI and controls were evaluated for the relationship between PET results and QSM values.

The results show a strong correlation between the density of amyloid-beta plaques and a load of iron deposition in the temporal and caudate nuclei, frontal, temporal, peritoneal, and occipital lobes in people with MCI. But this relationship was not seen in healthy people.

Finally, they concluded that iron accumulation could reflect brain dysfunction due to the deposition of amyloid-beta plaques and the risk of Alzheimer's disease [70].

In 2021, a study was conducted by PM Cogswell for this purpose.

Susceptibility in deep and lower gray nuclei, especially pallidum and putamen, was correlated with PET test results and associated with amyloid-beta deposits.

Contrary to the correlation found with iron and amyloid plaques associated with nerve damage in the deep nuclei, this relationship was insignificant in the cortex areas [8].

In 2020, Nicola Spotorno examined the relationship between iron and tau accumulation using magnetic resonance-based quantitative susceptibility mapping and tau-PET in 236 subjects with amyloid-b pathology.

Both voxel-wise and regional analyses showed a consistent association between differences in bulk magnetic susceptibility, which can be primarily ascribed to an increase in iron content and tau-PET signal in regions known to be affected by Alzheimer’s disease.

A significant relationship between quantitative susceptibility and tau-PET was more substantial in younger subjects [46].

## Sequence parameters and different QSM reconstruction steps algorithms

In this section, we prepared tables based on the material and methods of the selected articles.

In each research project, we have written sample information and parameters of the executed sequence (Tables 8, 9, 10, 11).

**Table 8:** Demographic information and MRI scan parameters related to Alzheimer's Disease research projects

Study	Au et al. [49]	Li et al. [48]
TR	45 ms	–
TR/TE	–	41.8/3.3 ms
Flip angle	20$^\circ$	20$^\circ$
Bandwidth	–	62.50 kHz
FOV	240 × 240 × 120 mm^3^	256 × 256 mm^2^
Matrix size	–	256 × 256
Slice thickness	–	1.0 mm
Slice numbers	–	124
TEs	8 echoes, TE1: 4.0 ms /*Δ*TE: 5.2 ms	16 echoes/TE spacing: 2.3 ms
Total time	5 min and 19 s	–
Sequence	3D fast-field echo\(FFE)	3D multi gradient-echo (mGRE)
Participants	13 Patients with early stage AD, 10 Patients with late stage AD, and 30 healthy subjects	22 Patients with AD, 22 Patients with MCI, 25 Patients with SCD, and 25 healthy subjects
MRI scanner system	3T MRI system (Philips Achieva TX, Best, The Netherlands) with an 8-channel head coil	3T MRI system (Discovery MR 750, GE Healthcare, Milwaukee, WI) equipped with a 32-channel phased-array coil

Study	Kim et al. [45]	Moon et al. [47]	Acosta-Cabronero et al. [44]
TR	43 ms		35 ms
TR/TE	–	37 ms/3.5 ms	–
Flip angle	20°	20°	*17*$^\circ$
Bandwidth	–	± 41.67 kHZ	50 Hz/pixel
FOV	220 × 198 mm^2^	240 × 240 mm^2^	
Matrix size	–	256 × 256	256 × 240
Slice Thickness	–	2.5 mm	2.0 mm
slice Numbers	–	56	72
TEs	TE_1_: 3.4/*Δ*TE: 6.0/ TE_7_: 39 ms	8 echoes/*Δ*TE: 4.09 ms	20 ms
Total Time	–	3 min and 32 s	7 min
Sequence	3D fast *field*-echo (FFE)	Susceptibility-weighted angiography sequence [SWAN]	Susceptibility-weighted-imaging (SWI)
Participants	19 patients with aMCI, 19 patients with mild and probable AD, and 19 healthy subjects	12 patients with VaD, 27 patients with AD, and 18 healthy subjects	8 Patients with early-stage probable AD
MRI scanner system	3T MRI system (Achieva, Philips Medical Systems, Best, The Netherlands)	3T MRI system (Signa HDxT; GE Medical Systems, Milwaukee, WI, USA) with an 8-channel head coil	3T MRI system (Siemens Trio 3T superconductive magnet with gradient coils)

Study	Cogswell et al. [8]	Gong [43]	Du et al. [5]
TR	28 ms	*250 *ms	22.9 ms
TR/TE	40/49 ms	–	–
Flip Angle	15°	*35*°	12°
Bandwidth	25.6 kHz	–	± 31.25 Hz/pixel
FOV	200 × 200 mm^2^	*19.2 *$\times$* 14.4 *$\times$* 9.6 *cm^3^	25.6 × 25.6 cm^2^
Matrix size	384 × 269	–	256 × 256
Slice thickness	1.8 mm	–	1.0 mm
Slice numbers	88	–	–
TEs	6.7, 10.6, 14.5, 18.4, and 22.4 ms	*TE1: 3.72//*Δ*TE: 5.52//TE10*: *53.36 *ms	3.2 ms
Total time	6:37 min	90 min	4 min and 24 ms
Sequence	3D-MEGRE	Multi-echo, 3D gradient echo (mGRE)	3D gradient-echo (GRE)
Participants	69 patients with MCI, 56 patients with amnestic dementia,and 296 healthy subjects	4 Pairs of transgenic mice with abnormal beta amyloid-aggregation (Tg-SwDI) and wild type	30 Patients with AD
MRI scanner Sys##tem	3T 3T MRI system (Siemens Prisma VE11C)	7T MRI system (Bruker BioSpec 70/20USR, Billerica, MA) with an Avance III system	3T MRI system (Discovery MR750 scanner; GE Medical Systems, USA)

**Table 9:** Demographic information and MRI scan parameters related to Parkinson’s Disease research projects

Study	Syam et al. [53]	Li [52]	Fedeli et al. [54]
TR	62.2 ms	–	36 ms
TR/TE	–	28/23 ms	–
Flip angle	15º	15°	20°
Bandwidth	–	–	–
FOV	–	230 × 230 × 180 mm^3^	–
Matrix size	–	256 × 256 × 180	512 × 512
Slice thickness	2 mm	–	–
Slice numbers	–	–	–
TEs	5 echoes Range: 5.7–29.5 ms	–	5/12/19/26/33 ms
Total time	–	–	–
Sequence	3D multi-echo gradient-echo (mGRE)	SWI with velocity-compensated 3D fast-field echo	3D spoiled multi-echo GRE sequences (mGRE)
Participants	26 Patients with PD, 27 Patients with PSP, and 26 healthy subjects	31 Non-demented PD patients, 10 patients with PDD and 27 healthy subjects	26 Patients 26 patients with primary atypical Parkinsonisms,and 49 patients with PD
MRI scanner system	3T MRI System (Discovery MR 750w, GE Healthcare, USA)	3.0T MRI System (Philips Achieva)	3T MRI System (Philips Achieva)

Study	Ide et al. [55]	Li et al. [73]	Shahmaei et al. [51]
TR	58.4 ms	32.80 ms	38 ms
TR/TE	–	–	–
Flip angle	15°	–	15°
Bandwidth	± 62.5 Hz	–	704 Hz/pixel
FOV	22 × 16.5 cm^2^	240 × 240 mm^2^	256 mm
Matrix size	320 × 416	320 × 320	256 × 256
Slice thickness	1.5 mm	–	1.5 mm
Slice numbers	1848	–	–
TEs	11 echoes TE_1_ = *4.5 ms* Time spacing = *5 ms*	11.00 ms	4, 41.8 ms
Total time	7 min 1 s	528 s	9 min
Sequence	3D multi-echo spoiled gradient echo (mGRE)	multi-echo GRE sequence (mGRE)	GRE T2*
Participants	19 Patients with PD and 41 healthy subjects	3 Patients with schizophrenia, 4 patients with dystonia, and 5 patients with Parkinson’s disease	30 Patients with PD and 15 healthy subjects
MRI scanner system	3T MRI System equipped with 8-channel phased-array coil	3T MRI System equipped with a 24-channel head coil	3T MRI System (Tim Trio Siemens Healthcare, Erlangen, Germany) With 32-channel coil

**Table 10:** Demographic information and MRI scan parameters related to other research projects

Study	Pu [50]
TR	60 ms
TR/TE	–
Flip angle	25°
Bandwidth	930 Hz/pixel
FOV	–
Matrix size	128 × 128 × 52
Slice thickness	–
Slice numbers	–
TEs	32 echoes, TE_1_: 2.4/ΔTE = 1.42 ms
Total time	40 min
Sequence	3D mGRE sequence with a bipolar readout gradient (mGRE)
Participants	16 Healthy adult macaques
MRI scanner system	3T wholebody MRI system. (MAGNETOM Trio, Siemens Healthcare A.G., Erlangen, Germany)

Study	Spincemaille et al. [33]	
TR	24.48 ms	24.55 ms
	45.08 ms	45.03 ms
TR/TE	–	
Flip angle	15 $^\circ$	15 $^\circ$
	15 $^\circ$	15 $^\circ$
Bandwidth	244.14	244.14
	244.14	244.14
FOV	220 × 176 mm^2^	220 × 176 mm^2^
	220 × 176 mm^2^	220 × 176 mm^2^
Matrix size	320 × 320 × 86	320 × 320 × 74
	320 × 320 × 86	320 × 320 × 74
Slice thickness	–	
Slice numbers	–	
TEs	5 echoes: 3.85/7.97/12.09/16.21 /20.33	5 echoes: 3.81/7.91/12.00/16.10/20.20
	10 echoes: 3.85/7.97/12.09/16.21/20.33/24.45/28.57/32.69/36.81/40.93	10 echoes: 3.81/7.91/12.00/16.10/20.20/24.29/28.39/32.48/36.58/40.68
Total time	3 min and 35 s	2 min and 51 s
	6 min and 36 s	5 min and 15 s
Sequence	3Dmultiple echo gradient echo (mGRE)	3Dmultiple echo gradient echo (mGRE)
Participants	10 healthy subjects	
MRI scanner system	3T MRI System (Discovery MR750, General Electric Healthcare)	Prototype 7T MRI System (MR950, Signa 7.0T, General Electric Healthcare,Waukesha, WI)

Study	Li et al. [34]	Sun and Wilman [32]	Wei et al. [31]
TR	40 ms	–	–
TR/TE	–	49/40 ms	50/40 ms
Flip angle	15$^\circ$	15$^\circ$	16°
Bandwidth	217 Hz/px	25.6 kHz	
FOV	224 × 224 × 140 mm^3^	230 × 207 × 136 mm^3^	230 × 230 × 132 mm^3^
Matrix size	–	–	128 × 128 × 66
Slice thickness	–	–	–
Slice numbers	–	–	–
TEs	6 echoes: TE_1_: 6/ΔTE: 6 ms	–	–
Total time	7 min and 19 s	5 min and 50 s	7 min and 30 s
Sequence	3D multi-echo gradient echo (mGRE)	standard gradient recalled echo (GRE) 3D-radiofrequency spoiled GRE sequence	standard flow-compensated 3D fast spoiled-gradient-recalled (SPGR)
Participants	10 Healthy subjects	6 Healthy subjects	7 Healthy subjects
MRI scanner system	3T MRI System (Philips Achieva scanner)	1.5T MRI System (Siemens Medical Solution, Erlangen, Germany)	3T MRI System (GE Healthcare, Waukesha, WI, USA)

Study	Spotorno et al. [46]
TR	24 ms
TR/TE	–
Flip angle	15$^\circ$
Bandwidth	490 Hz/pixel
FOV	–
Matrix size	–
Slice thickness	–
Slice numbers	–
TEs	5.00, 8.80, 12.60, 16.40 and 20.20 ms
Total time	3:54 min
Sequence	3D, multi gradient-echo pulse sequence (mGRE)
Participants	236 amyloid-*b*-positive subjects, 78 cognitively unimpaired, and 158 cognitively impaired patients
MRI scanner system	3T MRI System (Siemens Prisma 3 T scanner with a 64-channel receiver-coil array)

Study	Li et al. [35]		
TR	53 ms	25 ms	25 ms
TR/TE	–	–	–
Flip angle	–	–	–
Bandwidth	–	–	–
FOV	–	–	–
Matrix size	–	–	–
Slice thickness	–	–	–
Slice numbers	–	–	–
TEs	40 ms	17.5 ms	17.5 ms
Total time	–	–	–
Sequence	Gradient echo imaging (GRE)	Gradient echo imaging (GRE)	Gradient echo imaging (GRE)
Participants	114 Healthy subjects	336 Healthy subjects	173 Healthy subjects
MRI scanner system	3T MRI System (Siemens Prisma 3.0T scanner)	3T MRI System (Philips Ingenia 3.0T scanner)	3T MRI System (GE HDX 1.5T scanner)

**Table 11:** Algorithms for implementing each stage of QSM reconstruction based on the method of research projects reviewed in this study.

	Cogswell et al. [8]	Gong [43]	Kim et al. [45]	Moon et al. [47]	Acosta-Cabronero et al. [44]	Au et al. [49]	Li et al. [52]	Fedeli et al. [54]	Ide et al. [55]
QSM Compution	Sparse linear equations and least squares (LSQR) method	Using an iLSQR algorithm	Morphology Enabled Dipole Inversion (MEDI)	To calculate the quantitative susceptibility map (QSM)	Truncated k-space division method	Streaking artifact reduction for QSM (STAR-QSM)	L1-norm total-variationbased regularization algorithm	Streaking artifacts reduction (STAR) algorithm	Morphology enabled dipole inversion (MEDI)
Background Phase Removal	–	Spherical mean value filtering with an initial kernel width of 30 voxels and the kernel width decreasing toward the tissue boundary	Projection onto dipole *fi*elds (PDF)	By using projection onto the dipole field (PDF)		Variable spherical kernel size (V-SHARP) method	RE-SHARP	V-SHARP	
Phase Unwrapping	Laplacian-based	Using a Laplacian-based phase unwrapping algorithm		Performing nonlinear fit to the multi-echo data	The Laplacian-based method	Laplacian-based algorithm	Laplacian-based algorithm	Laplacian-based phase unwrapping	
Brain tissue mask Extraction	Align the TIV mask with the magnitude and phase GRE images	Using magnitude image			Brain extraction tool (BET)				

	Shahmaei et al. [51]	Pu et al. [50]	Spincemaille et al. [33]	Li et al. [34]	Sun and Wilman [32]	Li et al. [35]	Li et al. [73]
QSM Compution		Using fast STAR-QSM algorithm	The morphology-enabled dipole inversion (MEDI) algorithm	Modified structural feature based collaborative reconstruction algorithm (SFCR)	With regularization parameter of 5 $\times$ 10^–4^ and total variation technique	Truncated k-space division (TKD) based inverse filtering technique	Using streaking artifact reduction
Background Phase Removal	Using a high pass filter and a SHARP filter	V_SHARP method	The projection onto dipole field algorithm to yield the local field	V-SHARP method	Regularization enabled sophisticated harmonic artifact reduction for phase data (RESHARP)	The sophisticated harmonic artifact reduction (SHARP)	Using the V_SHARP method
Phase Unwrapping		Laplacian based phase unwrapping		Using Laplacian based	Laplacian based phase unwrapping	3D phase unwrapping algorithm (3DSRNCP) skipped for the simulated data too	Laplacian based phase unwrapping
Brain tissue mask Extraction	Brain extraction tool (BET)	Brain extraction tool (BET)	Brain extraction tool (BET)		Brain extraction tool (BET)	Brain extraction tool (BET)	Brain extraction tool (BET)

In the following, we collected the algorithms implemented in each stage of QSM reconstruction from these articles.

In the parts where the table is empty, the desired information is not explicitly mentioned.

These tables are a rich collection of information that can be used in selecting the method of further research.

The parameter tables of the first part are related to the method of articles related to AD, and the second part is related to Parkinson's disease.

Final tables are for other research projects such as deep brain stimulation (DBS) surgery targeting, evaluating an aging process or other items.

## Discussion

Based on past research projects, we know that microscopic or pathological changes such as iron or amyloid-beta plaques deposition in the deep nuclei of the brain precede morphological changes such as atrophy of various areas of the brain.

However, due to the lack of reliable biomarkers sensitive to these changes, the diagnosis can be made at an advanced stage and based on clinical findings.

Magnetic susceptibility is the innate and physical response of tissue to applying an external magnetic field that determines the components of the tissue.

Proteins are in the group of diamagnetic materials. The accumulation of amyloid-beta plaques, which are the main features of Alzheimer's disease, leads to an increase in the density of paired electrons and a change in the local magnetic susceptibility of the region (decreased tissue magnetic susceptibility).

On the other hand, iron is a group of paramagnetic materials. It causes positive changes in the magnetic susceptibility of tissues, so amyloid-beta plaques and the iron depositions in tissues have opposite effects on the magnetic susceptibility of tissues [1, 71].

QSM technique is a new and non-invasive method for clinical evaluations that has good sensitivity and specificity for diagnosing pathological changes in the brain and can be used in the early diagnosis of these disorders.

Two features of Alzheimer's disease are the presence of amyloid-beta plaques and tau proteins.

According to the results of studies, the presence of iron in these depositions causes their production and accumulation, and as a result, oxidative damage and neuronal death occur more frequently.

The accuracy of the QSM technique has been evaluated with various PET techniques, and good results have been presented. However, the correlation between the two techniques is more robust in the deep brain nuclei and young people.

Increased QSM in the putamen nucleus is one of the primary brain changes in the early stages of Alzheimer's disease, which is proportional to the degree of cognitive impairment and can be used as a suitable biomarker.

Also, correlation studies of QSM values and age in putamen nuclei and Globus pallidus are positive in Alzheimer's patients, and a negative correlation has been reported in the caudate nucleus [5, 47].

In individuals with MCI, this positive correlation is observed in the nuclei of the globus pallidus and hippocampus [5].

QSM values of red nuclei, substantia nigra, and globus pallidus nuclei can be used to diagnose Parkinson's disease early.

Unlike other cases, red nucleus QSM values are significantly reduced in patients with Parkinson's disease.

One of the exciting results of this study was the difference between people with Parkinson's dementia versus no with dementia; the bilateral hippocampus in patients with dementia has positive magnetic susceptibility values.

Finally, high levels of QSM in the nucleus of the globus pallidus can help distinguish patients with APPs, such as PSP, from healthy individuals with Parkinson's.

However, red nucleus magnetic susceptibility has a strong relationship with the severity of disorders in PSP patients.

In other neurodegenerative disorders, the QSM technique can be used to identify and start the treatment process early.

Such as examination of striatum structures in Huntington's disease, basal ganglia and brainstem in Wilson disease, motor cortex in Amyotrophic lateral sclerosis (ALS), cerebellar in Friedreich ataxia (FA), and habhabenular in major depression (Table 12).

**Table 12:** Correlation of QSM findings in the spectrum of neurodegenerative diseases for the introduction of pathological biomarkers

Type of cognitive disorder	The brain structure that is prone to changing QSM values	Description
Alzheimer's Disease	Putamen nucleus	It is an appropriate biomarker for diagnosing AD in its early stages
Degree of Cognitive Impairment	Caudate nucleus	Assessing the degree of cognitive impairment in AD and MCI (positive correlation)
Parkinson's Disease	Red nucleus, Substantia nigra, and Globus Pallidus nuclei	These nuclei QSM values can be used to diagnose and stage patients with Parkinson's disease
Degree of Clinical severity in Parkinson's Disease	Red nucleus,Subtania nigra, Globus Pallidus, and Hippocampus	These nuclei QSM values can be used to diagnose and stage patients with Parkinson's disease
Parkinson's disease dementia patients vs. non-demented patients with Parkinson's disease	Bilateral Hippocampus	Higher iron deposition in Parkinson's disease dementia patient's bilateral hippocampus
Atypical Parkinsonisms	Globus Pallidus	These nuclei QSM values can be used for early diagnosis and differentiation between APPs
Patients with progressive supranuclear palsy	Globus Pallidus	Patients with progressive supranuclear palsy (PSP) have higher magnetic susceptibility values in caudate, putamen, globus pallidus, and red nuclei compared to PD patients and control
Degree of Clinical severity in PSP	Red nucleus	QSM values can be used to diagnose and stage PSP patients
Huntington's Disease	Striatum	One of the most acute symptoms of Huntington's disease is an increase in iron depositions in the striatum, which causes free radicals and damage to neurons
Wilson disease	Basal Ganglia and Brainstem	The QSM technique shows increased susceptibility in the basal ganglia and brainstem of patients with Wilson disease
Amyotrophic lateral sclerosis(ALS)	Motor Cortex	abnormally high levels of iron in the motor cortex cause oxidative stress and the death of nerve cells
Friedreich ataxia (FA)	Cerebellar	A reduction in the size of the cerebellar
Major Depression	Habhabenular	One of the most acute symptoms of Major Depression is an increase in iron depositions in the habhabenular

In selecting the appropriate MRI scan sequence for QSM reconstruction, one should pay attention to the multi-echo nature of the sequence and preferably use GRE sequences for this purpose.

The most appropriate and widely used algorithms for each stage were identified based on previous research (Table 13).

**Table 13:** The most widely used algorithms of different stages of QSM reconstruction in research projects

Reconstruction stage	Generating Tissue Mask	Phase unwrapping	Background field removal	Solving the ill-posed inverse problem
Appropriate algorithm	BET toolbox in FSL	The Laplacian-based phase-unwrapping method	V_SHARP method	Morphology-enabled dipole inversion (MEDI)

Different methods are available for each step of QSM reconstruction. Still, the BET toolbox in FSL for tissue mask extraction, the Laplacian-based phase-unwrapping method for phase unwrapping, the V_SHARP method for the Background phase removal step, and finally, the morphology-enabled dipole inversion (MEDI) to compute the QSM and dipole inversion are the most used algorithms.

One of the most critical limitations of this research is the lack of access to clinical data of a suitable size, which can be achieved with more reliable results if research projects with this purpose are implemented in medical and research centers.

Perhaps the long execution time of this process and its related sequences or the Emerging of this technique are some of the factors involved in this issue.

## Limitations

One of the most critical limitations of this study was the small statistical size of any cognitive impairment group in the studies, which is better to use a more comprehensive database for more accurate analysis.

Another limitation is the lack of a clear standard for performing QSM reconstruction, which requires further research to optimize the parameters so that the results can be compared more reliably.

In addition to the above, there is no exact cut-off point for QSM values in each brain nucleus.

In fact, in any research, the researcher obtains these values based on the conditions and with different software, which requires extensive research to determine a specific standard in the use of cut-off points in studies.

## Conclusion

The QSM technique can be used to detect and differentiate neurodegenerative diseases with appropriate accuracy.

The high QSM values of the putamen nucleus are essential in the spectrum of disorders related to Alzheimer's disease.

The globus pallidus and red nuclei are important in the spectrum of disorders associated with Parkinson's disease and are prone to changes in magnetic susceptibility and QSM values.

Different algorithms have been used to perform different stages of QSM reconstruction, including BET for brain mask extraction, Laplacian-based method for phase unwrapping, V_SHARP toolbox for background field removal, and MEDI algorithm for final QSM reconstruction.

In general, QSM can be used clinically besides the gold standards methods, as long as sufficient data can be obtained to evaluate the method considerably.

It is generally suggested that updates to this research be written periodically, with larger datasets and using PET scan data.

Also, the evaluation of different kernels used in QSM reconstruction has not been evaluated in this study, which could become a valuable study.

## Data Availability

The datasets analysed during the current study are available in the [google scholar] repository, [https://scholar.google.com/]. And all data generated or analysed during this study are included in this published article [tables and figures in the paper].

## Abbreviations

AD: Alzheimer's disease
ALS: Amyotrophic lateral sclerosis
ALIC: Anterior limb of the internal capsule
BET: Brain extraction tool
CN: Caudal nucleus
DBS: Deep brain stimulation
EPI: Echo-planar imaging
FDRI: Field-dependent relaxation rate increase
GM: Gray matter
GP: Globus palidus
GPM: Globus pallidus medialis
HEIDI: Homogeneity-enabled incremental dipole inversion
LBV: Laplacian boundary value
mGRE: Multi-echo gradient echo
MoCA: Montreal cognitive assessment
MWF: Myelin water fraction
PDD: Parkinson's disease dementia
PLIC: Posterior limb of the internal capsule
PSP: Progressive supranuclear palsy
Pu: Putamen
RESHARP: Regularization enabled SHARP
ROIs: Region of interest
SFCR: Structural feature-based collaborative reconstruction
SN: Substantia nigra
SWI: Susceptibility-weighted imaging
tau-PET: Tau-positron emission therapy
QSM: Quantitative susceptibility mapping
WM: White matter
aMCI: Amnestic mild cognitive impairment
MD: Major depression
BG: Basal Ganglia
COSMOS: Susceptibility calculation through multidirectional sampling
DN: Dentate nucleus
FA: Friedreich ataxia
fQSM: Functional QSM
GMV: Gray matter volume
GPL: Globus Pallidus Lateralis
HARPARELLA: Harmonic phase removal using a laplacian operator
IC: Internal capsule
MEDI: Morphology-enabled dipole inversion
MMSE: Mini-Mental State Examination
MRI: Magnetic resonance imaging
PD: Parkinson's disease
PDF: Projection onto dipole fields
ppm: Part per million
PRISMA: Preferred reporting items for systematic reviews and meta-analyses
R_2_*: Relaxation rates
RN: Red nucleus
SD: Steepest descent
SHARP: Sophisticated harmonic artifact reduction for phase data
STN: Subthalamic nucleus
T_2_*WI: T_2_ * weighted imaging
TKD: Thresholded-K-space-division
VaD: Vascular dementia
